# An analytical expression for R50% dependent on PTV surface area and volume: A cranial SRS comparison

**DOI:** 10.1002/acm2.13168

**Published:** 2021-01-25

**Authors:** Dharmin D. Desai, E. L. Johnson, Ivan L. Cordrey

**Affiliations:** ^1^ Department of Radiation Oncology CHI Memorial Hospital Chattanooga TN USA; ^2^ Department of Radiation Medicine University of Kentucky Chandler Medical Center Lexington KY USA

**Keywords:** cranial SRS/SRT, dose drop‐off distance, PTV surface area, R50%_Analytic_, R50%

## Abstract

The intermediate dose spill for a stereotactic radiosurgery (SRS) plan can be quantified with the metric R50%, defined as the 50% isodose cloud volume (V_IDC50%_) divided by the volume of the planning target volume (PTV). By coupling sound physical principles with the basic definition of R50%, we derive an analytical expression for R50% for a spherical PTV. Our analytical expression depends on three quantities: the surface area of PTV (SA_PTV_), the volume of PTV (V_PTV_), and the distance of dose drop‐off to 50% (Δr). The value of ∆r was obtained from a simple set of cranial phantom plan calculations. We generate values from our analytical expression for R50% (R50%_Analytic_) and compare the values to clinical R50% values (R50%_Clinical_) extracted from a previously published SRS data set that spans the V_PTV_ range from 0.15 to 50.1 cm^3^. R50%_Analytic_ is smaller than R50%_Clinical_ in all cases by an average of 15% ± 7%, and the general trend of R50%_Clinical_ vs V_PTV_ is reflected in the same trend of R50%_Analytic_. This comparison suggests that R50%_Analytic_ could represent a theoretical lower limit for the clinical SRS data; further investigation is required to confirm this. R50%_Analytic_ could provide useful guidance for what might be achievable in SRS planning.

## 1 INTRODUCTION

A cranial stereotactic radiosurgery (SRS) plan should be highly conformal and have the steepest possible dose gradient outside of the planning target volume (PTV) to reduce complications associated with excessive radiation delivered to normal brain tissues as measured by the volume receiving 12 Gy^1^ or other intermediate dose threshold. Several dose gradient metrics have been designed to quantify the intermediate dose spill outside the PTV. These include gradient index (GI), gradient measure (GM), and R50%.^2^, ^3^, ^4^ The value of a given intermediate dose spill metric achievable in a clinical setting is likely a complex function of the size, shape, and location of the PTV in the cranium, as well as delivery geometry, treatment modality, and optimization performance. Based on analyses of clinical treatment plans, Goldbaum et al. and Ballangrud et al. have provided guidance on limiting values of the GI in cranial SRS planning utilizing the known PTV volume (V_PTV_).^5^, ^6^ Knowledge of this limit may be useful to the treatment planner as it provides a realistic goal to pursue in the optimization.

Wang et al. noted that the original Radiation Therapy Oncology Group (RTOG) protocols 90‐05 and 93‐05 make no mention of intermediate dose spill.^7^ However, the importance of intermediate dose spill, as measured by GI or R50%, in SRS/SRT plan evaluation is now widely recognized. Furthermore, two plans can have very similar high dose region conformity but have very different intermediate dose spill. The plan with the larger intermediate dose spill does more damage to surrounding tissue; thus, a smaller GI or R50% would yield less collateral damage. In this work, we examine the R50% metric to better understand what limits can be expected for R50% in high quality SRS/SRT plans.

Guidelines for intermediate dose spill metrics used in treatment planning tend to be phenomenological constructs, and limits so obtained are based on observations from large numbers of treatment plans. We have proposed a model‐based approach for the metric R50% that considers the physical characteristics V_PTV_ and PTV surface area (SA_PTV_). This approach allows for the derivation of an analytical form of R50% (R50%_Analytic_) that is based on physical principles. It is necessary, however, that this analytical methodology be validated against clinical data. At least one published study on cranial SRS does provide the necessary data for a meaningful comparison of R50%_Analytic_ to clinical data.^8^ Zhao et al. provided clear, tabulated data for a wide range of PTV volumes from 0.15 to 50.1 cm^3^. These clinical data sets are used to calculate R50% clinical values (R50%_Clinical_), which are directly compared to our predicted R50%_Analytic_ values in this paper. Note: A list of abbreviations is provided in the Appendix A.

## 2 MATERIALS AND METHODS

### 2.A R50%_Analytic_ derivation

**Fig. 1:**
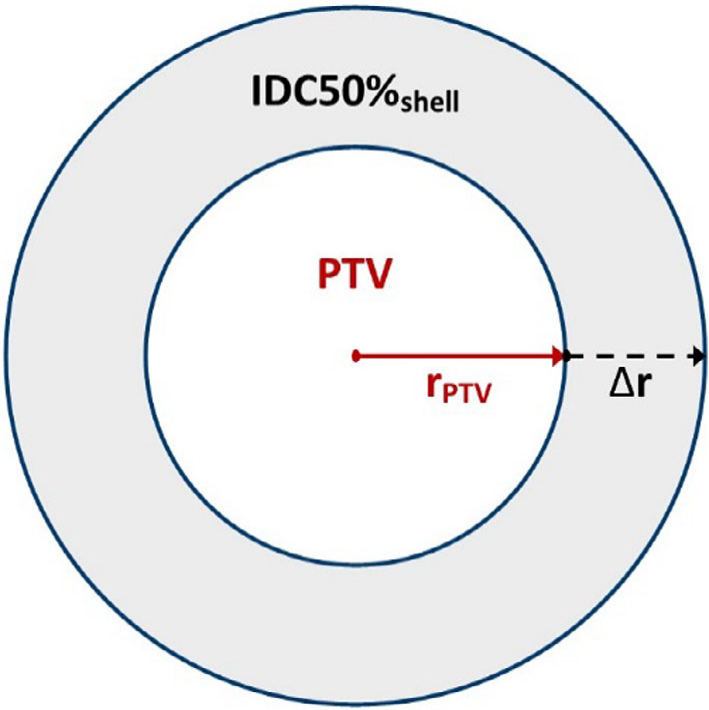
Plane through the center of the spherical volumes. Inner volume is the planning target volume (PTV). The shaded region is the spherical shell bounded by the 50% isodose cloud and the PTV surface area. ∆r is the radial thickness of the shell, as well as the distance of dose drop‐off from the edge of the PTV to 50%.

Consider a spherical PTV volume, V_PTV_, surrounded by a spherical shell that encloses the 50% isodose cloud volume (V_IDC50%shell_) as illustrated in Fig. 1. The sum of V_PTV_ and V_IDC50%shell_ is the total volume encompassed by the 50% isodose cloud (V_IDC50%_). R50% is defined as the ratio of the volume of the 50% Isodose Cloud to the volume of the PTV as follows:

(1) $$R50\%=\frac{V_{\text{IDC}50\%}}{V_{\text{PTV}}}=\frac{V_{\text{PTV}}+V_{\text{IDC}50\%\text{shell}}}{V_{\text{PTV}}}=1+\frac{V_{\text{IDC}50\%\text{shell}}}{V_{\text{PTV}}}$$

Furthermore, we determined an exact value of V_IDC50%shell_ by integrating the spherical differential shell volume, 4πr^2^dr, from r = r_PTV_ to r = r_PTV_ + ∆r.

(2) $$\begin{array}{c} V_{\text{IDC}50\%\text{shell}}=\int_{r_{\text{PTV}}}^{r_{\text{PTV}}+\Delta r}4\pi r^{2}\text{dr}=\frac{4}{3}\pi \left[{\left(r_{\text{PTV}}+\Delta r\right)}^{3}‐r_{\text{PTV}}^{3}\right]=\\ 4\pi r_{\text{PTV}}^{2}\Delta r\left[1+\frac{\Delta r}{r_{\text{PTV}}}+\frac{1}{3}{\left(\frac{\Delta r}{r_{\text{PTV}}}\right)}^{2}\right] \end{array}$$

Given that SA_PTV_ $=4\pi r_{\text{PTV}}^{2}$ and combining Eqs. (1) and (2), the resulting analytical form of R50% can be expressed as:

(3) $$R50{\%}_{\text{Analytic}}=1+\frac{{\text{SA}}_{\text{PTV}}}{V_{\text{PTV}}}\Delta r\left[1+\left(\frac{\Delta r}{r_{\text{PTV}}}\right)+\frac{1}{3}{\left(\frac{\Delta r}{r_{\text{PTV}}}\right)}^{2}\right]$$

Equation (3) is a form of R50% for a spherical volume. We identify the three components within the square brackets of Eq. (3) as zeroth order, first order, and second order terms, respectively. This complete expression is an extension of previous work that only used the zeroth order term and, as expected, significantly improves agreement for smaller PTV volumes.^9^, ^10^

### 2.B Δr determination

One additional requirement of this analytical approach is an estimate of the dose drop‐off to 50% parameter, ∆r, which cannot be calculated from first principles at this time. However, it is possible to obtain realistic estimates of ∆r from treatment planning studies. Note that Δr is likely different for different treatment modalities (i.e., Gamma Knife, Cyber Knife, and SRS capable Linacs) and should be determined for each technology.

In our spherical model, the dose drop‐off parameter Δr is the value of linear distance from the edge of the PTV to the outer edge of IDC50%_shell_ as shown in Fig. 1 and is taken as isotropic.

**Fig. 2:**
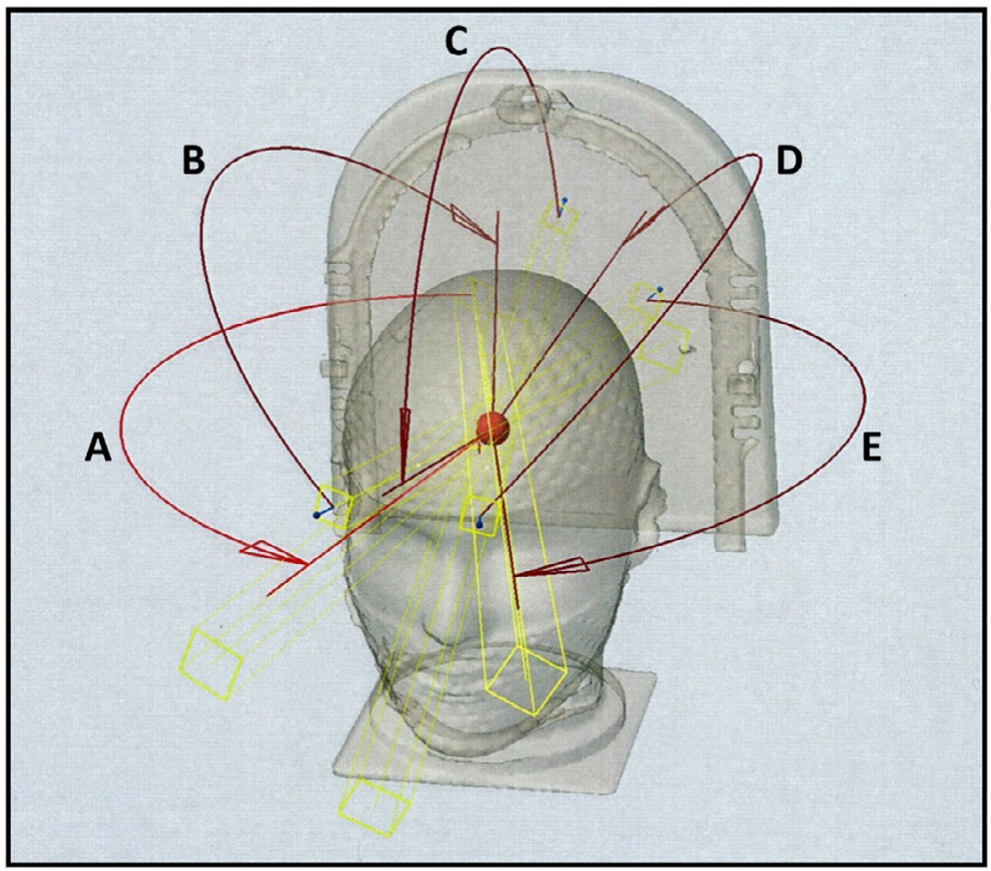
The five hemi‐arcs beam arrangement for determination of Δr. This three‐dimensional (3D) view of the IROC head phantom shows the beam delivery geometry used for the phantom plans used to determine Δr for a series of nine spherical planning target volumes. Each red curve in the figure represents the path of an arc around the cranium using the Varian IEC scale. For couch angles 355° (A), 315° (B), and 270° (C), the arcs span 195° to 345°. For couch angles 45° (D) and 5° (E), the arcs span 15° to 165°.

To experimentally determine a value of Δr for the R50%_Analytic_ calculations, we utilized a treatment planning CT of the IROC SRS Head Phantom (IROC Houston QA Center, Houston, TX) as the anthropomorphic phantom model. Nine spherical PTVs were created in the center of the cranium with volumes ranging from 0.19 to 44 cm^3^. Treatment planning was performed on an Eclipse radiation treatment planning system (RTPS) using the photon optimizer PO v15.6 with a final calculation via the AAA v 15.6 algorithm on a 1 mm calculation grid size. All plans were created for a Varian TrueBeam STx with a 120 leaf HD MLC and used volumetric modulated arc therapy (VMAT, RapidArc) techniques. The delivery geometry employed in this study to determine Δr used five hemi‐arcs spanning 150° arc angles at five couch angles as shown in Fig. 2. This geometry is both clinically reasonable and highly conformal for a central cranial tumor because it uses nearly a full 2π solid angle. The prescription for PTVs with a volume ≤ 3 cm^3^ was 18 Gy in one fraction with 99% of the V_PTV_ receiving the dose; the prescription for PTVs with a volume > 3 cm^3^ was 27 Gy in three fractions with 99% of V_PTV_ receiving the prescription dose (D99% volumetric prescription). One could also use a percent isodose line (PIDL) prescription to achieve the same volumetric PTV coverage as one achieves with the volumetric prescription.^11^ Ultimately, we just need 99% of the PTV volume covered by the prescription dose consistently for all plans that determine Δr such that CI is very nearly 1.0. Eclipse NTO (Normal Tissue Objective) was used in conjunction with three dose control shells (inner control shell, middle control shell, and outer control shell) as described by Clark et al. to directly limit the dose spill outside the PTV, in accordance with standard clinical practices.^12^ Alternatively, one could use other dose limiting shell techniques.^13^ We sought the minimum value of Δr one could obtain clinically in ideal circumstances. The quality of these phantom plans can be seen from the parameters given in Table 1.

**Table 1:** Summary of treatment planning properties obtained from the IROC SRS head phantom study to determine the value of Δr.

V_PTV_ (cm^3^)	r_PTV_ (cm)	CI_RTOG_	HI_RTOG_	GM (cm)	r_IDC50%_ (cm)	Δr (cm)	PIDL
0.19	0.36	1.18	1.80	0.20	0.57	0.22	57.3
0.55	0.51	0.99	1.26	0.25	0.76	0.25	80.8
0.99	0.62	1.04	1.38	0.27	0.90	0.28	72.8
1.96	0.78	1.04	1.36	0.30	1.09	0.31	83.2
2.96	0.89	1.03	1.31	0.34	1.23	0.34	78.5
3.97	0.98	1.04	1.27	0.35	1.34	0.36	79.6
6.93	1.18	0.99	1.22	0.40	1.58	0.40	85.4
20.45	1.70	0.99	1.21	0.52	2.22	0.52	88.7
43.99	2.19	0.99	1.21	0.65	2.83	0.64	91.7
	Ave CI_RTOG_	1.03					
	Std Dev	0.06					

CI_RTOG_ is the conformity index, and HI_RTOG_ is the homogeneity index. All plans are normalized volumetrically to D99% (99% of the PTV volume receives 100% of the prescription dose). The equivalent PIDL is determined by matching the coverage of the D99% prescription. Δr values are calculated from the difference of r_IDC50%_ and r_PTV_, assuming both volumes are spherical. Note the Eclipse GM values are nearly identical to Δr.

**Fig. 3:**
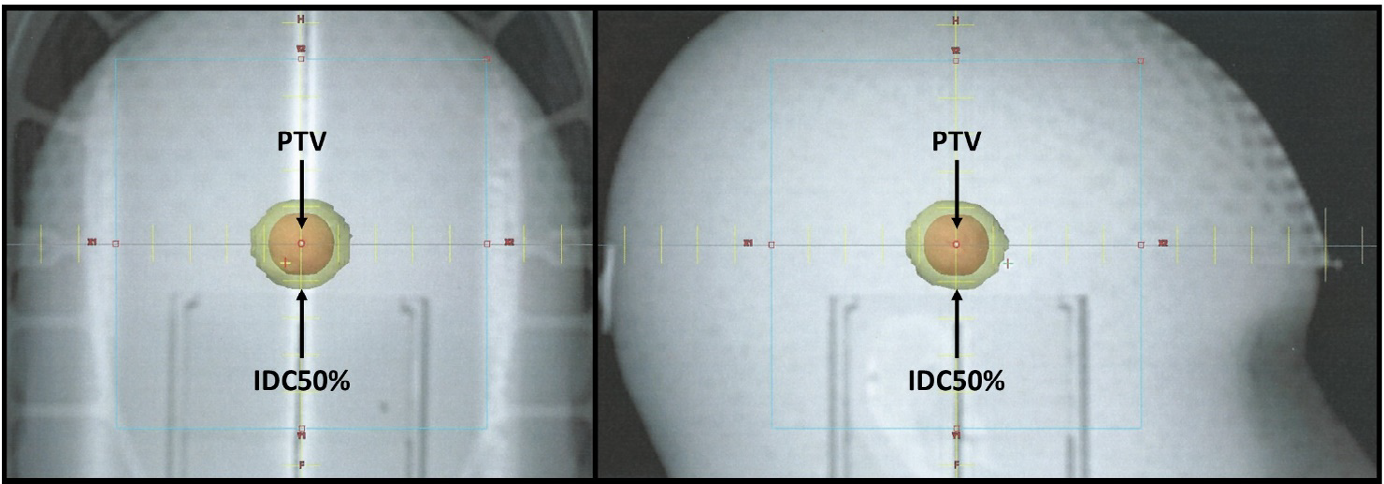
Typical results for the phantom study to determine ∆r. The diagram shows an AP DRR and a right lateral DRR that display the position and size of the PTV (solid orange shape) and IDC50% (transparent yellow shape) within the cranium. The distance from the edge of the PTV and the outer edge of IDC50% is Δr. The volume of the PTV is 3 cm^3^. Note that the IDC50% is very nearly spherical.

Since a highly noncoplanar delivery geometry coupled with a spherical PTV was chosen, the resulting dose distribution is reasonably isotropic and can be assumed spherical. This nearly spherical dose distribution can be clearly seen in Fig. 3 as the transparent yellow isodose cloud of 50% of the prescription dose (IDC50%) surrounding the solid orange PTV. This distribution bears a marked similarity to Fig. 1 used in the derivation of R50%_Analytic_. Thus, it becomes simple to extract a value of Δr for each phantom PTV as follows:

(4) $$\Delta r=r_{\text{IDC}50\%}‐r_{\text{PTV}}$$

**Fig. 4:**
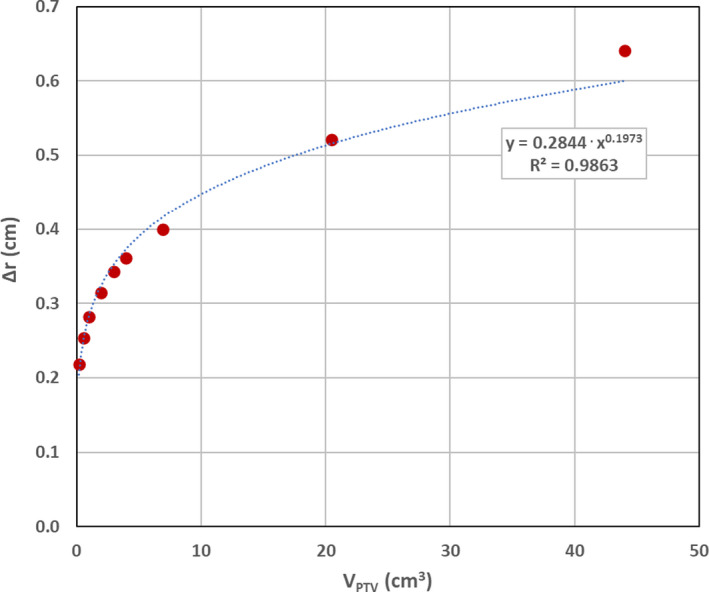
Phantom study derived Δr as a function of V_PTV_. A good fit is obtained with the power law function shown.

Based on the values of Δr obtained from the phantom study, a power law fit was generated (Microsoft Excel) for Δr as a function of V_PTV_ as shown in Fig. 4.

The resulting power law expression for Δr, in units of cm, is:

(5) $$\Delta r=0.2844\times V_{\text{PTV}}^{0.1973}$$

where V_PTV_ is measured in cm^3^.

As can be seen in Table 1, the GM values reported by Eclipse for these spherical volumes are nearly identical to the Δr values obtained from Eq. (4). This should not be surprising since GM is defined as the difference, in centimeters, of the equivalent sphere radii of V_IDC50%_ and V_IDC100%_ (r50%_eq_ and r100%_eq_, respectively).^7^ Thus,

(6) $$\text{GM}=r50{\%}_{\text{eq}}‐r100{\%}_{\text{eq}}$$

By comparison, for a perfectly conformal plan (CI = 1.0), V_IDC100%_ is identical to and spatially coincident with V_PTV_. Thus, for a spherical PTV, r100%_eq_ = r_PTV_. Furthermore, if IDC50% is assumed to be spherical, r50%_eq_ = r_IDC50%_. Therefore, it is reasonable to assume that for nearly spherical volumes, the GM values obtained from Eclipse can be considered equivalent to Δr. For simplicity, Δr was only considered as a function of V_PTV_.

### 2.C Comparison methodology

To validate the clinical relevancy of R50%_Analytic_, we compared values generated from Eq. ((3), (4), (5), (6), (7), (8)) to R50%_Clinical_ values obtained from a published data set. Zhao et al. performed a retrospective analysis of 30 clinical cases and investigated an optimal prescription isodose line that yields the steepest dose fall‐off (smallest GI) outside the PTV for cranial SRS plans. While R50% values are not directly presented in the retrospective analysis, clinical values for GI and CI_RTOG_ values are given for all 30 cases. Given the following definitions of GI and CI_RTOG_,

(7) $$\text{GI}=\frac{V_{\text{IDC}50\%}}{V_{\text{IDC}100\%}}$$

and

(8) $${\text{CI}}_{\text{RTOG}}=\frac{V_{\text{IDC}100\%}}{V_{\text{PTV}}}$$

R50% can be seen as the product of Eqs. (7) and (8).

(9) $$R50\%=\frac{V_{\text{IDC}50\%}}{V_{\text{PTV}}}=\frac{V_{\text{IDC}50\%}}{V_{\text{IDC}100\%}}\times \frac{V_{\text{IDC}100\%}}{V_{\text{PTV}}}=\text{GI}\times {\text{CI}}_{\text{RTOG}}$$

Using this approach, the data of Zhao et al. will yield the equivalent R50% to be used for comparison.

## 3 RESULTS

Table 2 contains V_PTV_, CI_RTOG_, and GI values directly transcribed from Zhao et al., values calculated from the clinical data, and the subsequently generated R50%_Analytic_ values. The parameter r_PTV_ was calculated using an assumption that PTV is spherical, and thus, it is an equivalent sphere radius of the PTV. SA_PTV_ is the surface area of the equivalent sphere PTV. R50%_Clinical_ was obtained by multiplying the clinical CI_RTOG_ and GI values provided by Zhao et al. [Eq. (9)].

**Table 2:** Clinical data and comparison of R50%_Analytic_ values to R50%_Clinical_ values.

V_PTV_^(a)^ (cm^3^)	r_PTV_^(c)^ (cm)	SA_PTV_^(b)^ (cm^2^)	CI_RTOG_^(a)^	GI^(a)^	R50%_Clinical_^(b)^	Δr (cm)	R50%_Analytic_	%Diff of R50% Values
0.15	0.33	1.37	1.59	3.87	6.15	0.20	4.05	−34.26
0.21	0.37	1.71	1.30	3.50	4.55	0.21	3.85	−15.47
0.37	0.45	2.49	1.61	3.14	5.06	0.23	3.54	−29.88
0.44	0.47	2.80	1.27	3.07	3.90	0.24	3.46	−11.25
0.48	0.49	2.96	1.30	3.19	4.15	0.25	3.42	−17.55
0.53	0.50	3.17	1.32	3.06	4.04	0.25	3.37	−16.49
0.61	0.53	3.48	1.23	3.00	3.69	0.26	3.31	−10.31
0.75	0.56	3.99	1.24	2.90	3.60	0.27	3.22	−10.46
1.30	0.68	5.76	1.21	2.75	3.33	0.30	3.00	−9.83
1.80	0.75	7.15	1.33	2.76	3.67	0.32	2.88	−21.48
2.10	0.79	7.93	1.25	2.62	3.28	0.33	2.83	−13.61
2.60	0.85	9.14	1.28	2.70	3.46	0.34	2.76	−20.18
3.10	0.90	10.28	1.14	2.57	2.93	0.36	2.70	−7.74
4.20	1.00	12.59	1.08	2.51	2.71	0.38	2.61	−3.67
4.70	1.04	13.57	1.20	2.48	2.98	0.39	2.58	−13.34
4.80	1.05	13.76	1.22	2.55	3.11	0.39	2.57	−17.29
6.10	1.13	16.14	1.15	2.41	2.77	0.41	2.51	−9.56
6.90	1.18	17.52	1.15	2.47	2.84	0.42	2.47	−12.91
7.30	1.20	18.20	1.16	2.48	2.88	0.42	2.46	−14.52
7.80	1.23	19.02	1.16	2.45	2.84	0.43	2.44	−14.08
9.50	1.31	21.69	1.22	2.68	3.27	0.44	2.39	−26.83
11.40	1.40	24.49	1.05	2.39	2.51	0.46	2.35	−6.42
12.60	1.44	26.18	1.11	2.44	2.71	0.47	2.32	−14.16
14.10	1.50	28.22	1.06	2.39	2.53	0.48	2.30	−9.25
18.80	1.65	34.19	1.12	2.36	2.64	0.51	2.24	−15.43
21.30	1.72	37.15	1.14	2.42	2.76	0.52	2.21	−19.93
27.30	1.87	43.84	1.27	2.13	2.71	0.55	2.16	−20.21
34.40	2.02	51.14	1.07	2.31	2.47	0.57	2.11	−14.50
41.70	2.15	58.14	1.06	2.29	2.43	0.59	2.08	−14.42
50.10	2.29	65.71	1.07	2.24	2.40	0.62	2.04	−14.71
							Ave %Diff	−15.32
							Std Dev	6.63

Values shown are actual and calculated parameters from Zhao et. al. SA_PTV_ values were calculated assuming spherical PTVs in the Zhao et al. data. Also shown are values of Δr and R50%_Analytic_ obtained from Eqs. (4) and (3), respectively. ^(a)^values given by Zhao et al. ^(b)^values calculated from Zhao et al. ^(c)^value calculated from Zhao et al. based on spherical PTV assumption.

**Fig. 5:**
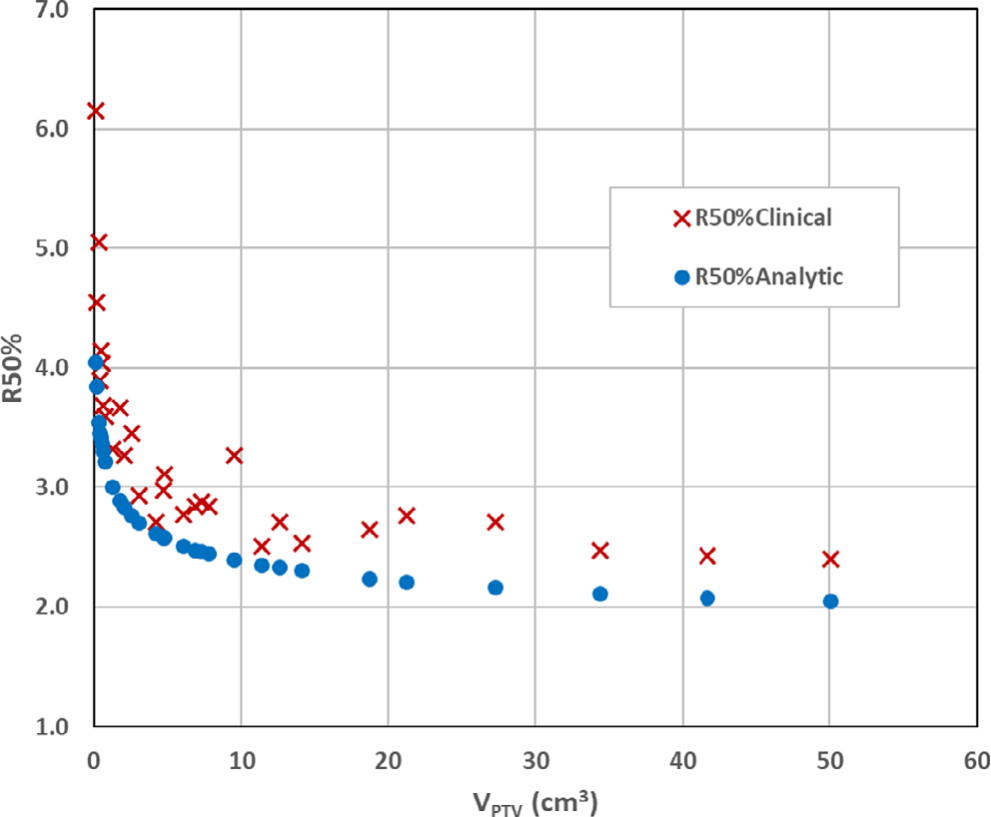
Comparison of R50%_Clinical_ and R50%_Analytic_ as functions of the V_PTV_ in the range from 0.15 to 50.1 cm^3^. This is a graphical representation of the data in Table 2. The R50%_Clinical_ values are extracted from the clinical study of Zhao et al. The values of R50%_Analytic_ are calculated from Eq. (3). Note that the general trend of R50%_Clinical_ as a function of V_PTV_ is reflected in the same trend of R50%_Analytic_. Furthermore, the R50%_Clinical_ values are consistently larger than the R50%_Analytic_ values.

Table 2 also displays the %Difference between the values of R50%_Clinical_ and R50%_Analytic_. R50%_Analytic_ values are uniformly smaller than R50%_Clinical_ values by an average of 15% ± 7%. A quick observation confirms that for smaller PTV volumes the R50%_Clinical_ values are significantly larger than the R50%_Analytic_ results obtained from Eq. (3). As an example, for the smallest PTV volume (0.15 cm^3^), R50%_Clinical_ is 34.3% larger than R50%_Analytic_. These data are also shown graphically in Fig. 5, which indicates the larger R50%_Clinical_ values over the PTV volume range included in this study.

## 4 DISCUSSION

It can be readily seen that R50%_Analytic_ values are consistently lower than the corresponding R50%_Clinical_ data (Fig. 5). Consideration of the treatment planning conditions of Zhao et al. may provide a basis for a reasonable explanation of the differences observed. The clinical data presented by Zhao et al. are a composite of situations influenced by a wide range of conditions: unique prescription doses, diverse sizes and shapes, various locations in the brain, and variable proximity to different organs at risk among other restrictions. The distance of dose drop‐off from PTV surface to 50% (Δr) is likely affected by some of these conditions. In contrast, consider the ideal conditions assumed in the derivation of Eq. (3). For simplicity, isotropic dose drop‐offs from PTV surface to 50% were assumed around spherical PTVs, which implies a 4π delivery geometry. In most realistic scenarios, the treatment of cranial targets can achieve a 2π delivery geometry for a Linac‐based SRS delivery. Clinical PTVs, however, are not ideal spheres, and dose drop‐offs are not perfectly isotropic around the PTV. Also, clinical considerations of organs at risk in proximity of the PTV were not included in the R50%_Analytic_ model. As a result, the R50%_Analytic_ model, as indicated by Eq. (3), should be considered as a theoretical lower limit of R50% for intracranial targets.

We measured Δr in a simple planning study of spherical targets of varying volumes. Our planning study used VMAT (RapidArc) delivery. A similar study could be done to determine Δr using dynamic conformal arc therapy (DCAT), and the values of Δr so obtained could be different. The data provided by Zhao et al. for the replan of clinical cases were done using DCAT delivery. If the Δr was larger as a function of V_PTV_ using DCAT delivery, the agreement with the data provided by Zhao et al. would improve. However, our goal in this work was to provide the minimum achievable R50% as described by R50%_Analytic_. We chose to determine Δr using VMAT techniques because VMAT delivery of SRS/SRT is rapidly gaining popularity, particularly for multiple target cases.^6^, ^11^

There are other ways to measure or estimate Δr and similar quantities. We used a simple planning study and the GM functionality built into Eclipse. Sung and Choi use proprietary software to determine cumulative dose gradient index (cDGI), a metric of their creation similar to Δr in the case of the cDGI for the 50% of prescription dose (cDGI50%).^14^ They determine the cDGI50% for a 3 cm diameter spherical target (V_PTV_ = 14.14 cm^3^) to be cDGI50% = 5.98 mm. Our empirical formula for Δr [Eq. (5)] for that same volume yields Δr = 4.80 mm, which is comparable to the value of cDGI50%. R50%_Analytic_ will be a larger value if one uses cDGI50% as the estimate for Δr. Zhang et al. propose yet another novel metric they call dose‐dropping speed (DDS).^15^ Dose‐dropping speed certainly has relationship to Δr and shows similar dependence on V_PTV_, which they describe in terms of PTV diameter. In fact, to compare values for 1/DDS to our Δr values, one finds they are within 0.1 mm for a 0.9 cm^3^ target and within 1.4 mm for a 61.6 cm^3^ target, with the Zhang et al. determined values of 1/DDS being the larger values.

In our work, we do not propose a new metric but rather a way to predict the minimum value of an established metric, R50%, for an SRS/SRT case based on three parameters: V_PTV_, SA_PTV_, and Δr. Because Δr cannot be calculated from first principles at this time, we measure Δr for the case of spherical targets. Yet, the value of Δr is not the primary focus of this work. Our primary focus is testing the equation R50%_Analytic_ against the clinical data provided by Zhao et al.

Goldbaum et al. noted that a group of plans with very similar PTV volumes produced a wide range of R50% values. They hypothesized that the increase in R50% could be related to variations in SA_PTV_ but were not able to quantify the relationship. Although this current study only considered spherical volumes, the dependence on SA_PTV_ is explicit in Eq. (3), and conceptually, this analytic model should be able to account for variations in SA_PTV_. In fact, the model would predict larger R50% values for targets with increased SA_PTV_ to V_PTV_ ratios, which is consistent with the suppositions in Goldbaum et al. In previous work, it was quantitatively shown that an increase in the SA_PTV_ to V_PTV_ ratio leads to an increase in R50% values.^9^ For any given volume, the shape that corresponds to the smallest surface area is a sphere,^16^ and the assumption of a spherical PTV with an isotropic dose drop‐off is central to the construction of our analytic equation for R50% [Eq. (3)]. This reflects an ideal case, and therefore, it would be reasonable to argue that the analytical equation yields the smallest possible R50% (the R50% lower limit). Zhao et al. provided V_PTV_ values for their study but did not provide SA_PTV_ data. However, this is not unexpected since commercial treatment planning systems do not include surface area as part of the structure statistics as they report (like V_PTV_). Without available surface area information, we assumed a spherical PTV (smallest surface area) and calculated SA_PTV_ from the provided V_PTV_ values; the calculated SA_PTV_ values were then used in Eq. (3) to generate R50%_Analytic_. The actual clinical PTV shapes in the data of Zhao et al. are likely to have some nonspherical character.

At lower V_PTV_ values, a larger difference is seen between R50%_Clinical_ and R50%_Analytic_ (Fig. 5), which indicates that caution should be taken when evaluating clinical values of R50% at low PTV volumes. Zhao et al. suggested that, for small PTV volumes, dose drop‐off is extremely sensitive to location, target shape, and beam settings and discussed the limitation of treatment planning systems to accurately compute dose for small targets. Our analytic form does not suffer from those clinical and technical challenges, and thus, it is a reasonable assumption that, for a certain V_PTV_, the smallest theoretical R50% value is expressed by Eq. (3). This prediction could be used as a guide for the treatment planner to consider, among other factors, when progressing through the plan optimization. A set of PTVs of a given volume could have different shapes and, thus, different surface areas. Equation (3) clearly shows that a larger surface area PTV should have a larger R50%. As such, knowing the SA_PTV_ and recognizing that a larger surface area guarantees a higher R50% value can be useful at the onset of the treatment planning process. Based on the comparison results with Zhao et al., a plan with R50% within 15% of the R50%_Analytic_ would be a plan with excellent intermediate dose spill.

It is possible that R50%_Analytic_ could be used for automated planning or artificial intelligence planning systems that seek to control intermediate dose spill.^13^ As such, R50%_Analytic_ would be used as the target or goal R50% of the automated planning. R50%_Analytic_, as expressed in Eq. (3), may not be achievable in all circumstances, but as stated above, a plan within 15% of the R50%Analytic is a plan with excellent intermediate dose spill.

Understanding intermediate dose spill when multiple PTVs are optimized simultaneously using a single isocenter is not a trivial task. It depends on several factors: relative locations and sizes of PTVs with respect to one another (e.g., a large PTV in close proximity to a much smaller PTV), plan delivery geometry, plan optimization performance, etc. There is no easy or straight forward way to account for an increase in R50% of a PTV due to its location with respect to another PTV. Drawing from comments of Bohoudi et al. and Goldbaum et al. stating that their results obtained for intermediate dose spill around single cranial targets should apply to multiple cranial target cases as well,^5^, ^17^ we expect R50%_Analytic_ to perform well in predicting the theoretical minimum R50% for individual PTVs in multiple target cranial SRS/SRT cases. This will need to be confirmed by further investigation.

## 5 CONCLUSION

An analytical expression for R50% was derived for the special case of spherical volumes. The expression appears to provide a lower limit of R50% when compared to peer‐reviewed, clinical data. We surmise that SA_PTV_ plays an important role in the determination of the R50% value ultimately achievable in treatment planning. Further research is needed to establish the role of SA_PTV_ for other PTV shapes in the determination of treatment planning outcomes. Research is also needed to establish methods for obtaining Δr and investigate additional determining factors beyond V_PTV_.

## CONFLICT OF INTEREST

No conflict of interest.

## APPENDIX A

### ABBREVIATIONS

Table A1 contains definitions for abbreviations used throughout this article.

**Table A1:** List of abbreviations with definitions.

Abbreviation	Definition
cDGI	Cumulative dose gradient index
CI_RTOG_	RTOG conformity index
D99%	99% of PTV volume covered by 100% of prescription dose
DDS	Dose‐dropping speed
GI	Gradient index
GM	Gradient measure
HI_RTOG_	RTOG homogeneity index
IDC	Isodose cloud
IDC50%	50% (of prescription dose) isodose cloud
IDC50%shell	Distance from the edge of the planning target volume to the edge of the 50% isodose cloud
IDC100%	100% (of prescription dose) isodose cloud
NTO	Normal tissue objective; Instructs the optimizer to limit dose to non‐target volumes
OAR	Organs at risk
PIDL	Prescription isodose line
PTV	Planning target volume
Δr	Distance of dose drop‐off from the edge of the planning target volume to 50% dose
r_IDC50%_	Radius of the 50% isodose cloud
r_PTV_	Radius of the planning target volume
r50%_eq_	Equivalent sphere radius of the volume of the 50% isodose cloud
r100%_eq_	Equivalent sphere radius of the volume of the 100% isodose cloud
R50%	Ratio of the volume of the 50% isodose cloud to the volume of the planning target volume
R50%_Analytic_	Value of R50% generated from our analytical expression
R50%_Clinical_	Value of R50% calculated from clinical data
RTPS	Radiation treatment planning system
SA_PTV_	Surface area of the planning target volume
SRS	Stereotactic radiosurgery
SRT	Stereotactic radiotherapy
V_IDC50%_	Volume of the 50% isodose cloud
V_IDC50%shell_	Volume of the 50% isodose cloud minus the volume of the planning target volume
V_IDC100%_	Volume of the 100% isodose cloud
V_PTV_	Volume of the planning target volume
VMAT	Volumetric modulated arc therapy

## References

[1] Minniti G , Clarke E , Lanzetta G , et al. Stereotactic radiosurgery for brain metastases: analysis of outcome and risk of brain radionecrosis. Radiat Oncol. 2011;6:1–9. 21575163 10.1186/1748-717X-6-48 PMC3108308

[2] Paddick J , Lippitz B . A simple dose gradient measurement tool to complement the conformity index. J Neurosurg. 2006;105:194–201. 10.3171/sup.2006.105.7.194 18503356

[3] Wagner TH , Bova FJ , Friedman WA , Buatti JM , Bouchet LG , Meeks SL . A simple and reliable index for scoring rival stereotactic radiosurgery plans. Int J Radiat Oncol Biol Phys. 2003;57:1141–1149. 14575847 10.1016/s0360-3016(03)01563-3

[4] Videtic GMM , Hu C , Singh AK , et al. A randomized phase 2 study comparing 2 stereotactic body radiation therapy schedules for medically inoperable patients with stage I peripheral non‐small cell lung cancer: NRG Oncology RTOG 0915 (NCCTG N0927). Int J Radiat Oncol Biol Phys. 2015;93:757–764. 26530743 10.1016/j.ijrobp.2015.07.2260 PMC4744654

[5] Goldbaum DS , Hurley JD , Hamilton RJ . A simple knowledge‐based tool for stereotactic radiosurgery pre‐planning. J Appl Clin Med Phys. 2019;20:97–108. 10.1002/acm2.12770 PMC6909177 31743563

[6] Ballangrud Å , Kuo LC , Happersett L , et al. Institutional experience with SRS VMAT planning for multiple cranial metastases. J Appl Clin Med Phys. 2018;19:176–183. 29476588 10.1002/acm2.12284 PMC5849827

[7] Wang D , DeNittis A , Hu Y . Strategies to optimize stereotactic radiosurgery plans for brain tumors with volumetric‐modulated arc therapy. J Appl Clin Med Phys. 2020;21:45–51. 32043810 10.1002/acm2.12818 PMC7075387

[8] Zhao B , Jin JY , Wen N , et al. Prescription to 50–75% isodose line may be optimum for linear accelerator based radiosurgery of cranial lesions. J Radiosurg SRBT. 2014;3:139–147. PMC5675486 29296395

[9] Desai DD , Cordrey IL , Johnson EL . A physically meaningful relationship between R50% and PTV surface area in lung SBRT. J Appl Clin Med Phys. 2020;21:47–56. 10.1002/acm2.12964 PMC7497922 32725674

[10] Desai DD , Johnson EL , Cordrey IL . An analytical expression for R50% dependent on PTV surface area and volume: a lung SBRT comparison. J Appl Clin Med Phys. 2020;21:278–282. 10.1002/acm2.13026 PMC7700934 32996668

[11] Xu Y , Ma P , Xu Y , Dai J . Selection of prescription isodose line for brain metastases treated with volumetric modulated arc radiotherapy. J Appl Clin Med Phys. 2019;20:63–69. 10.1002/acm2.12761 PMC6909111 31833642

[12] Clark GM , Popple RA , Prendergast BM , et al. Plan quality and treatment planning technique for single isocenter cranial radiosurgery with volumetric modulated arc therapy. Pract Radiat Oncol. 2012;2:306–313. 24674169 10.1016/j.prro.2011.12.003

[13] Rossi L , Romero AM , Milder M , de Klerk E , Breedveld S , Heijmen B . Individualized automated planning for dose bath reduction in robotic radiosurgery for benign tumors. PLoS One. 2019;14:e0210279. 30726214 10.1371/journal.pone.0210279 PMC6364873

[14] Sung K , Choi YE . Dose gradient curve: a new tool for evaluating dose gradient. PLoS One. 2018;13:e0196664. 29698471 10.1371/journal.pone.0196664 PMC5919624

[15] Zhang Q , Zheng D , Lei Y , et al. A new variable for SRS plan quality evaluation based on normal tissue sparing: the effect of prescription isodose levels. Br J Radiol. 2014;87:20140362. 25226047 10.1259/bjr.20140362 PMC4207160

[16] Kleiner B . An isoperimetric comparison theorem. Invent Math. 1992;108:37–47.

[17] Bouhoudi O , Bruynzeel AME , Lagerwaard FJ , Cuijpers JP , Slotman BJ , Palacios MA . Isotoxic radiosurgery planning for brain metastases. Radiother Oncol. 2016;120:253–257. 27212141 10.1016/j.radonc.2016.05.001

